# Supplementary material for: Imaging NRF2 activation in non-small cell lung cancer with positron emission tomography
Source: Nat Commun. Author manuscript; Available in PMC 2024 Dec 24. (PMC11652680; doi:10.1038/s41467-024-54852-4)
Supplement: Supplementary data [file EMS201905-supplement-Supplementary_data.pdf]

Supplementary Information: Imaging NRF2 activation in non-small cell lung cancer with positron emission tomography

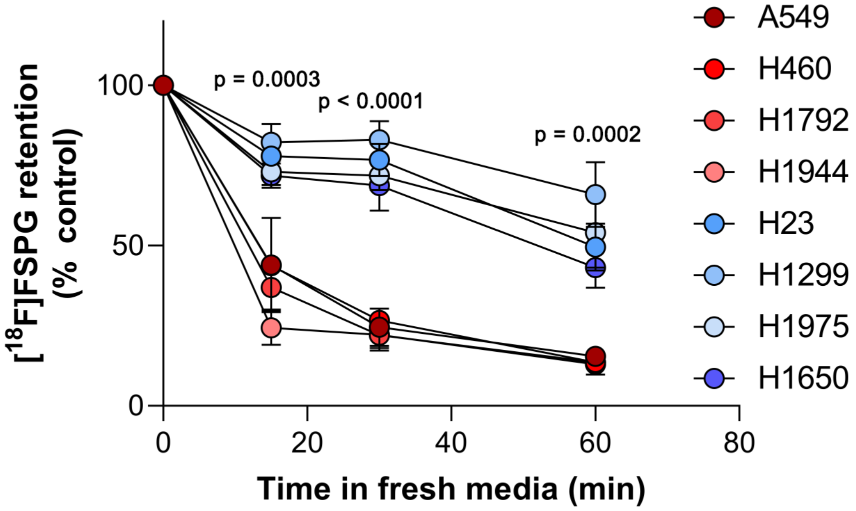

Supplementary Figure 1. Time course of [^{18}F]FSPG efflux across NRF2-high (red) and NRF2-low NSCLC cells (blue). Data are presented as mean ± SD from n = 3 independent experiments. Comparisons between groups (NRF2-high vs. NRF2-low cells) for each time point were made using an unpaired two-tailed Student's t-test. Source data are provided as a Source Data file.

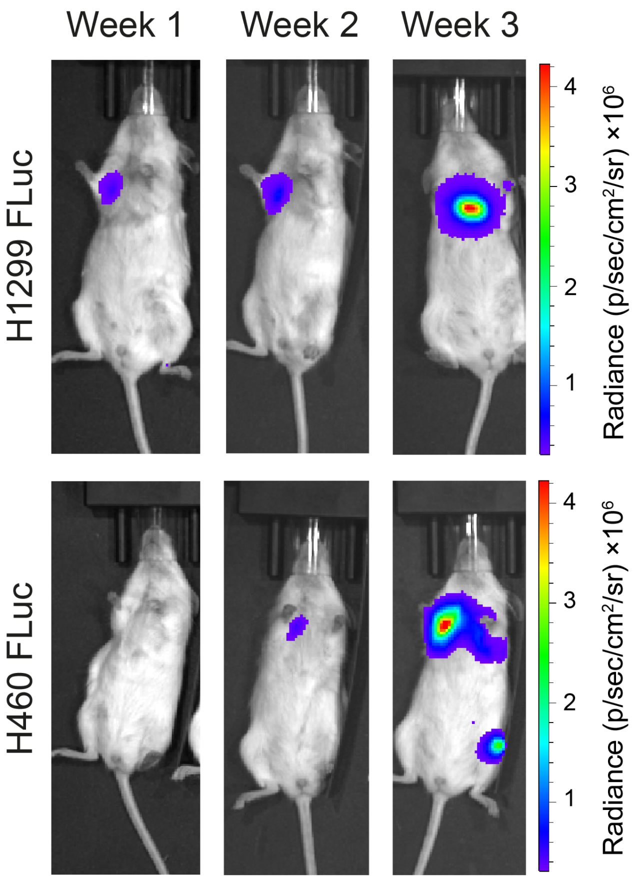

Supplementary Figure 2. Representative BLI from mice orthotopically implanted with H460 FLuc and H1299 FLuc tumour cells.

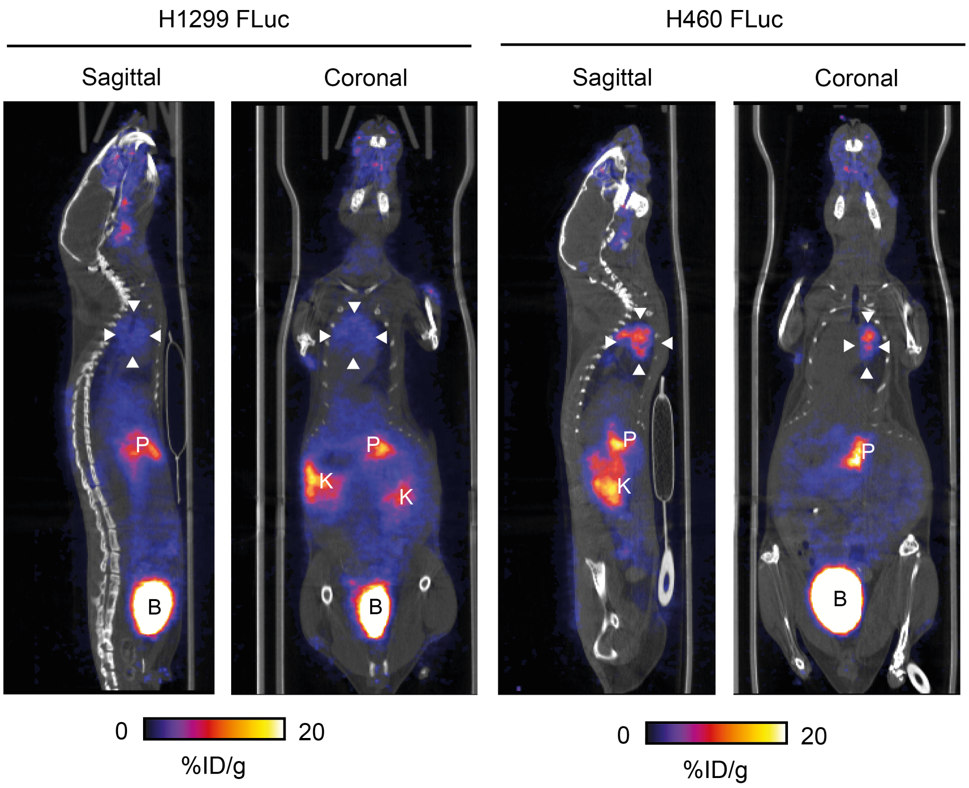

Supplementary Figure 3. Representative single slice PET/CT images from mice orthotopically implanted with H460 FLuc and H1299 FLuc tumour cells. Arrowheads denote the tumour. K, kidney; P, pancreas; B, bladder.

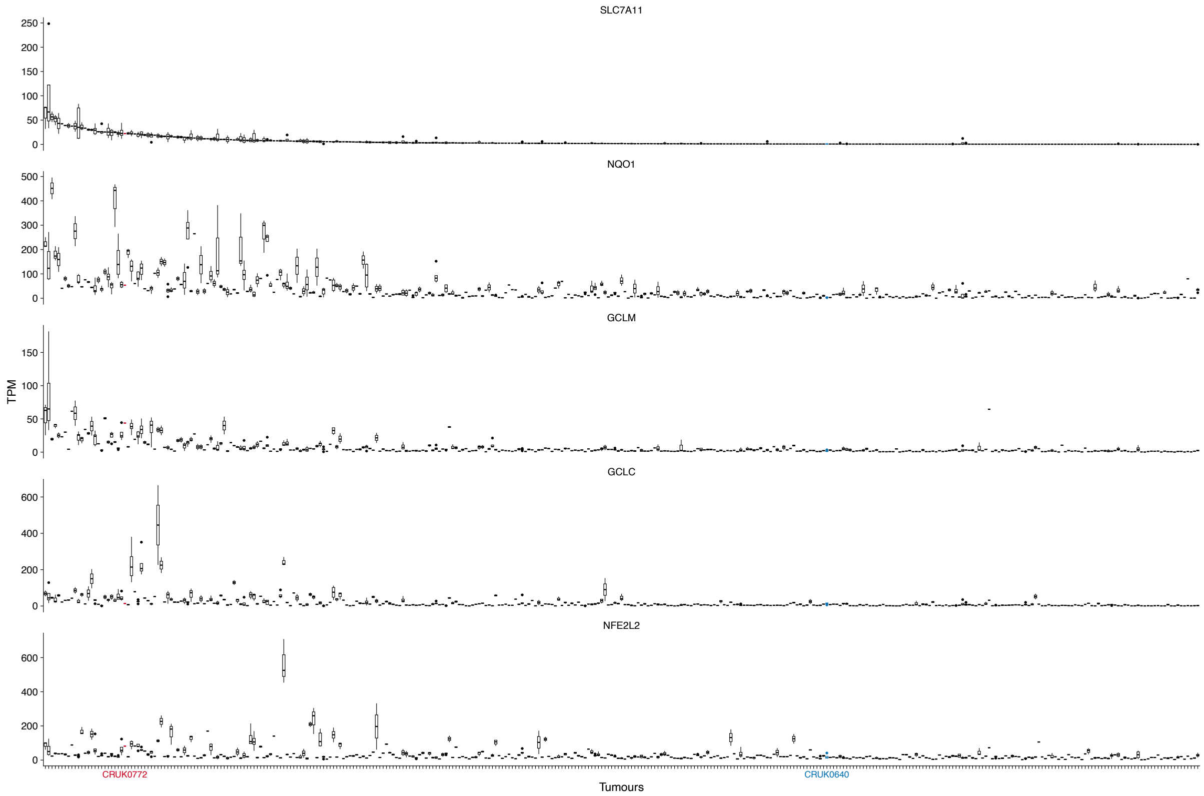

Supplementary Figure 4. Multi-region bulk RNA sequencing data from 882 samples from 349 NSCLC tumours from 344 patients. Plots show expression (transcripts per million) of *SLC7A11, NQO1, GCLM, GCLC and NFE2L2.* Box plots show the lower and upper quartiles (box limits) and the median (centre line). The length of whiskers is 1.5 × interquartile range and outlier points outside this range are shown. Tumours are ordered by *SLC7A11* expression. CRUK0772 (red) and CRUK0640 (blue) are indicated.

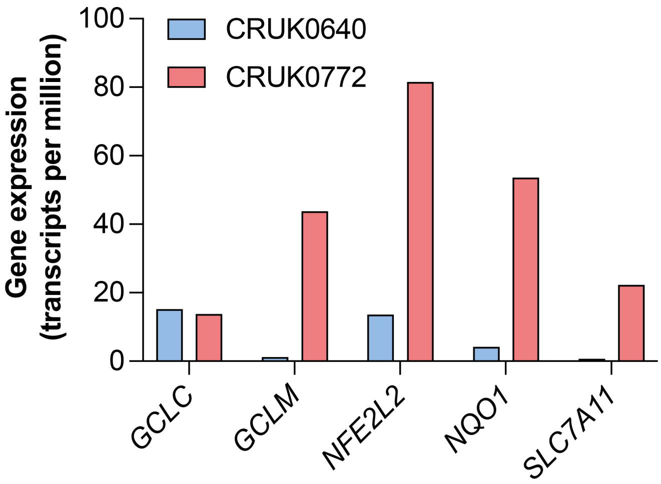

Supplementary Figure 5. NRF2-regulated gene expression in CRUK0640 and CRUK0772 tumour resection samples from TRACERx421 RNA sequencing data. Data are from two individual patients. Source data are provided as a Source Data file.

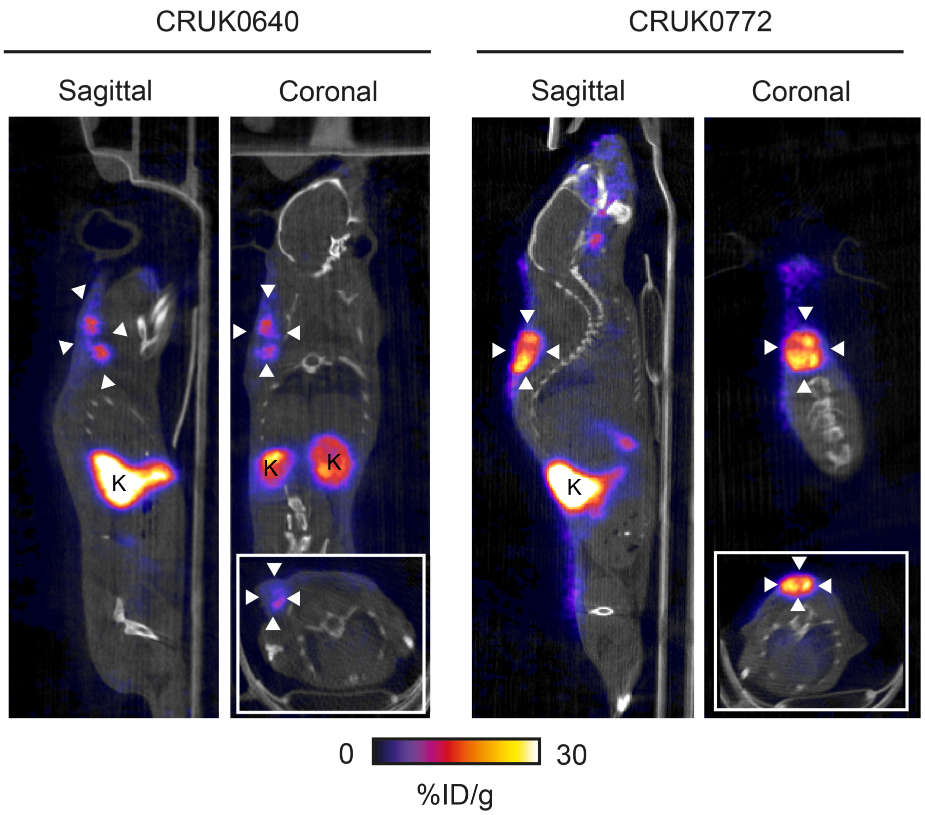

Supplementary Figure 6. Representative single slice PET/CT images from mice bearing CRUK0640 or CRUK0772 patient-derived xenografts. The axial view is shown as an insert. Arrowheads denote the tumour. K, kidney.

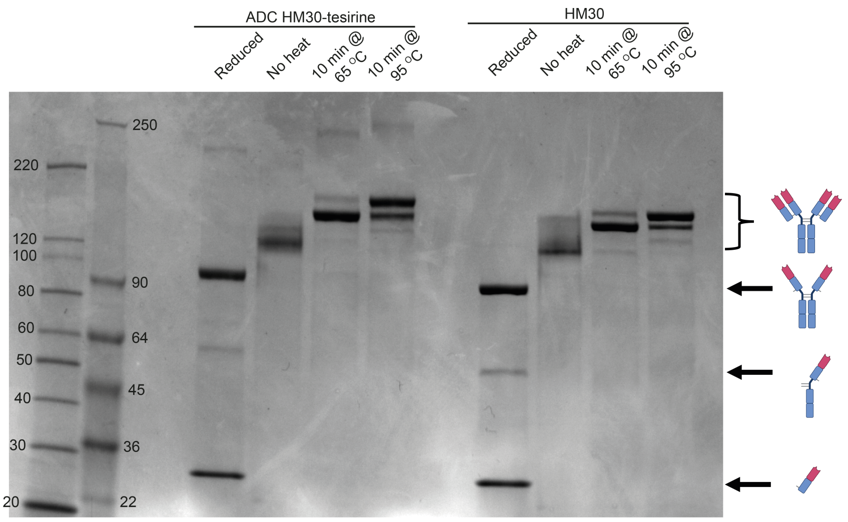

Supplementary Figure 7. HM30-tesirine and unconjugated HM30 monoclonal antibody SDS-PAGE profile with a range of pre-treatment conditions. Antibody schematics created with BioRender.com released under a Creative Commons Attribution-NonCommercial-NoDerivs 4.0 International license, https://creativecommons.org/licenses/by-nc-nd/4.0/deed.en

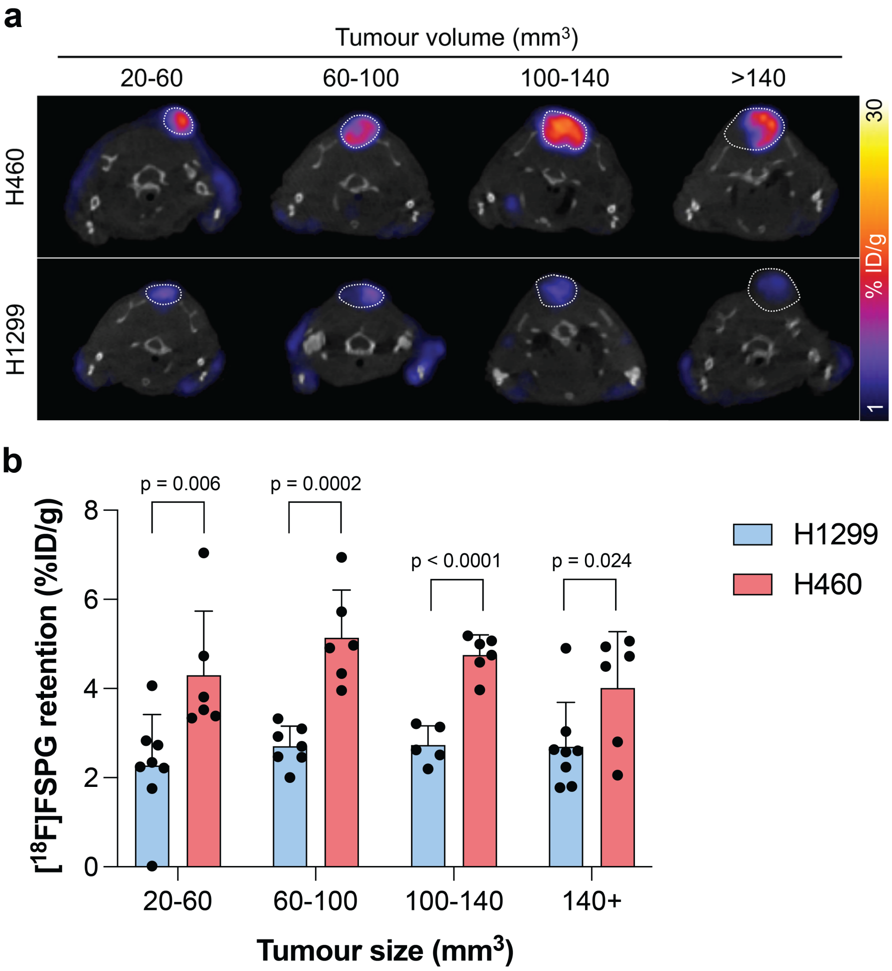

Supplementary Figure 8. [^{18}F]FSPG can distinguish NRF2-high from NRF2-low-expressing tumours across a range of tumour volumes. **a** Representative axial [^{18}F]FSPG PET/CT images of 40-60 min summed activity in mice bearing subcutaneous H460 (NRF2-high) and H1299 (NRF2-low) tumours of varying size. Dashed white lines indicate the tumour. **b** Quantification of [^{18}F]FSPG tumour retention. Data are presented as mean ± SD from n = 5-8 mice. Comparisons were made using an unpaired two-tailed Student's t-test. For (**b**) source data are provided as a Source Data file.

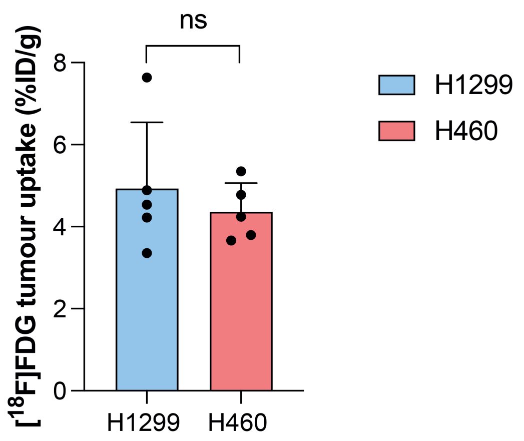

Supplementary Figure 9. [^{18}F]FDG cannot distinguish NRF2-high from NRF2-low-expressing tumours. Quantification of [^{18}F]FDG tumour retention in mice bearing H460 (NRF2-high) and H1299 (NRF2-low) subcutaneous tumours. ns, not significant. Data are presented as mean ± SD from n = 5 mice. Comparisons were made using an unpaired two-tailed Student's t-test. Source data are provided as a Source Data file.

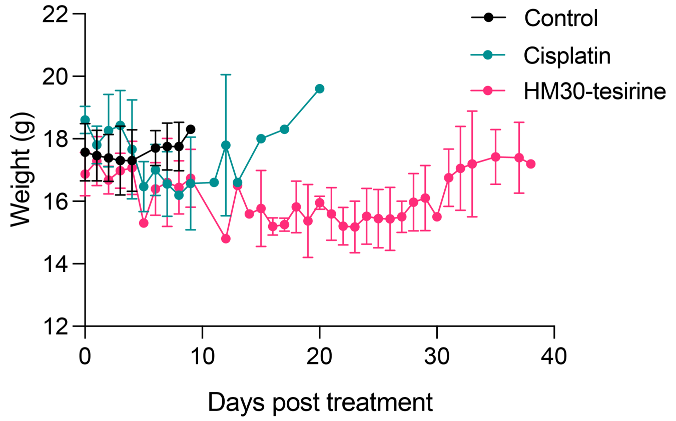

Supplementary Figure 10. Body weight from H460 tumour-bearing mice following treatment with vehicle control, cisplatin, and HM30-tesirine. Data are presented as mean ± SD from n = 5-6 mice. Source data are provided as a Source Data file.

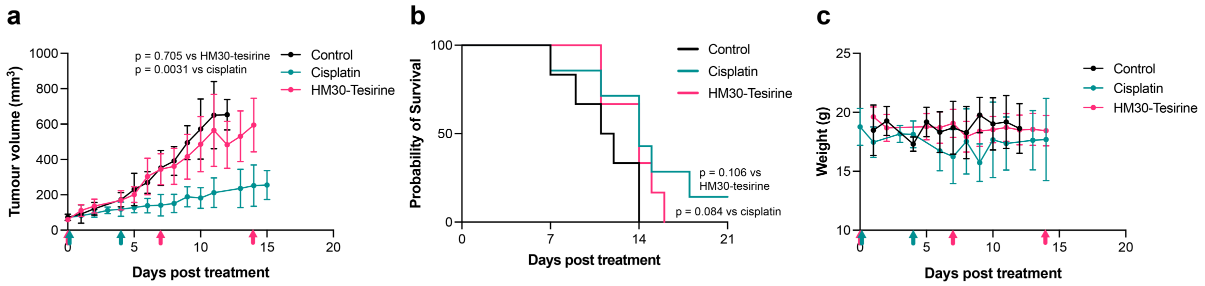

Supplementary Figure 11. HM30-tesirine has no efficacy in NRF2-low H1299 tumours. Anti-tumour activity (**a**), survival benefit (**b**) and animal weights (**c**) of control (saline treated), cisplatin treated, and HM30-tesirine treated mice. For (**a, c**) data are presented as mean ± SD from n = 6-7 mice. The arrows under the x-axis of (**a, c**) represent treatment cycles for cisplatin (green) and HM30-tesirine (red). For (**a**) comparisons were made on day 11 using a one-way ANOVA with followed by t-tests multiple comparison correction (Tukey method). For (**b**), statistics were analysed with a log-rank (Mantel–Cox) test. To control the family-wise error rate in multiple comparisons, crude p values were adjusted by the Holm–Bonferroni method. Source data are provided as a Source Data file.
