# Supplementary material for: Imaging NRF2 activation in non-small cell lung cancer with positron emission tomography
Source: Nat Commun. 2024 Dec 17;15:10484. doi: 10.1038/s41467-024-54852-4 (PMC11652680; doi:10.1038/s41467-024-54852-4)
Supplement: Supplementary file 1 [file 41467_2024_54852_MOESM3_ESM.pdf]

nature portfolio

Corresponding author(s): Timothy H. Witney

Last updated by author(s): Oct 22, 2024

Reporting Summary

Nature Portfolio wishes to improve the reproducibility of the work that we publish. This form provides structure for consistency and transparency in reporting. For further information on Nature Portfolio policies, see our Editorial Policies and the Editorial Policy Checklist.

Statistics

For all statistical analyses, confirm that the following items are present in the figure legend, table legend, main text, or Methods section.

n/a	Confirmed	
☐	☒	The exact sample size (*n*) for each experimental group/condition, given as a discrete number and unit of measurement
☐	☒	A statement on whether measurements were taken from distinct samples or whether the same sample was measured repeatedly
☐	☒	The statistical test(s) used AND whether they are one- or two-sided<br>*Only common tests should be described solely by name; describe more complex techniques in the Methods section.*
☒	☐	A description of all covariates tested
☐	☒	A description of any assumptions or corrections, such as tests of normality and adjustment for multiple comparisons
☐	☒	A full description of the statistical parameters including central tendency (e.g. means) or other basic estimates (e.g. regression coefficient) AND variation (e.g. standard deviation) or associated estimates of uncertainty (e.g. confidence intervals)
☐	☒	For null hypothesis testing, the test statistic (e.g. *F*, *t*, *r*) with confidence intervals, effect sizes, degrees of freedom and *P* value noted<br>*Give P values as exact values whenever suitable.*
☒	☐	For Bayesian analysis, information on the choice of priors and Markov chain Monte Carlo settings
☒	☐	For hierarchical and complex designs, identification of the appropriate level for tests and full reporting of outcomes
☒	☐	Estimates of effect sizes (e.g. Cohen's *d*, Pearson's *r*), indicating how they were calculated

Our web collection on statistics for biologists contains articles on many of the points above.

Software and code

Policy information about availability of computer code

Data collection	PET imaging data was acquired on a Mediso NanoScan PET/CT system.
Data analysis	Imaging data was analysed on VivoQuant and imunohistochemical images assessed in ImageJ. All other data performed using GraphPad Prism.

For manuscripts utilizing custom algorithms or software that are central to the research but not yet described in published literature, software must be made available to editors and reviewers. We strongly encourage code deposition in a community repository (e.g. GitHub). See the Nature Portfolio guidelines for submitting code & software for further information.

Data

Policy information about availability of data

All manuscripts must include a data availability statement. This statement should provide the following information, where applicable:

- Accession codes, unique identifiers, or web links for publicly available datasets
- A description of any restrictions on data availability
- For clinical datasets or third party data, please ensure that the statement adheres to our policy

All data are available in the article and its Supplementary materials. Source data are provided with this paper.

Research involving human participants, their data, or biological material

Policy information about studies with human participants or human data. See also policy information about sex, gender (identity/presentation), and sexual orientation and race, ethnicity and racism.

Reporting on sex and gender	This information has not been collected or used in this research.
Reporting on race, ethnicity, or other socially relevant groupings	This information has not been collected or used in this research.
Population characteristics	This information has not been collected or used in this research.
Recruitment	N/A
Ethics oversight	Ethical approval to generate patient-derived xenografts used in this study was granted through the TRACERx clinical study (REC reference: 13/LO/1546; NCT01888601).

Note that full information on the approval of the study protocol must also be provided in the manuscript.

Field-specific reporting

Please select the one below that is the best fit for your research. If you are not sure, read the appropriate sections before making your selection.

☒ Life sciences ☐ Behavioural & social sciences ☐ Ecological, evolutionary & environmental sciences

For a reference copy of the document with all sections, see nature.com/documents/nr-reporting-summary-flat.pdf

Life sciences study design

All studies must disclose on these points even when the disclosure is negative.

Sample size	No statistical methods were used to determine sample size. Three independent experiments were used for most experiments based on manufacturers recommendations for use of commercially available reagents/assays or based on our previously published work (DOI:10.7150/thno.63237). A minimum of seven lesions from three or more mice were assessed for in vivo imaging studies. Animal and lesion numbers are reported in the figures for clarity.
Data exclusions	In Figure 7e, tumour volumes for surviving mice where excluded when less than three mice remained in the treatment or control arm to alow for statistical analyses to be performed between groups. The exclusion criteria was pre-established. No other data exclusions were performed.
Replication	A minimum of three independent experiments were acquired for all cell-based work.
Randomization	Mice for imaging and treatment studies were randomised into size-matched cohorts.
Blinding	Investigators were not blinded during data collection or analysis.

Reporting for specific materials, systems and methods

We require information from authors about some types of materials, experimental systems and methods used in many studies. Here, indicate whether each material, system or method listed is relevant to your study. If you are not sure if a list item applies to your research, read the appropriate section before selecting a response.

Materials & experimental systems

n/a	Involved in the study	
☐	☒	Antibodies
☐	☒	Eukaryotic cell lines
☒	☐	Palaeontology and archaeology
☐	☒	Animals and other organisms
☐	☒	Clinical data
☒	☐	Dual use research of concern
☒	☐	Plants

Methods

n/a	Involved in the study	
☒	☐	ChIP-seq
☐	☒	Flow cytometry
☒	☐	MRI-based neuroimaging

Antibodies

Antibodies used	xCT, Cell Signaling Technology, 1291, lot 4, 1:1000<br>xCT, abcam, ab37185, 1:1000<br>NRF2, Cell Signaling Technology, 12721, lot 10, 1:1000<br>NQO1, Cell Signaling Technology, 62262, 1:1000<br>Actin, Cell Signaling Technology, 4967, lot 13, 1:1000<br>Vinculin, Sigma-Aldrich, V9264, 1:1000<br>Horseradish peroxidase anti-rabbit IgG secondary antibody , Cell Signaling Technology, 7074, 1:2000
Validation	All antibodies used in this study are commercially available and have been validated by the manufacturer and in previous studies.

Eukaryotic cell lines

Policy information about cell lines and Sex and Gender in Research

Cell line source(s)	Human NSCLC lines A549, H460, H1944, H1792, H23, H1299, H1975 and H1650 were purchased from LGC Ltd. H460 FLuc were purchased from PerkinElmer. H1299 FLuc were purchased from AMS biotec. A549 NRF2 knockout (KO), A549 KO-restored (KO-R), H1299 T80K and H1299 PLX317 were previously described (DOI: 10.7554/eLife.45572).
Authentication	All cell lines were authenticated by Short Tandem Repeat (STR) profiling by the provider.
Mycoplasma contamination	Cell lines were tested monthly for mycoplasma infection (Eurofins) and confirmed to be mycoplasma free.
Commonly misidentified lines<br>(See ICLAC register)	No commonly misidentified cell lines were used in this study.

Animals and other research organisms

Policy information about studies involving animals; ARRIVE guidelines recommended for reporting animal research, and Sex and Gender in Research

Laboratory animals	Female NOD/SCID/IL2Rg-/- (NSG) mice and LSL-KrasG12D/+; p53flox/flox; LSL-Nrf2D29H/+ mice on a mixed C57BL/6 genetic background were used. Mice enrolled in the study were aged between 6 and 9 weeks.
Wild animals	N/A
Reporting on sex	This information has not been collected or used in this research.
Field-collected samples	N/A
Ethics oversight	All animal experiments performed in the U.K. were in accordance with the United Kingdom Home Office Animal (Scientific Procedures) Act 1986 (project: PP9982297) and received local Animal Welfare and Ethical Review Body (AWERB) approval. For GEMM studies, Mice were housed and bred following the ethical regulations and approval of the University of South Florida Institutional Animal Care and Use Committee (protocol numbers: IS00005814M and IS00003893R). All animals were housed in Tecniplast Greenline Sealsafe IVC cages, fed standard rodent chow, received water ad libitum, and were housed in a facility with a 12 h light cycle at 20-24°C and 45-65% humidity.

Note that full information on the approval of the study protocol must also be provided in the manuscript.

Clinical data

Policy information about clinical studies

All manuscripts should comply with the ICMJE guidelines for publication of clinical research and a completed CONSORT checklist must be included with all submissions.

Clinical trial registration	This study contains retrospective analysis of existing clinical data. Details of the original study (NCT01888601) are reported in Martinez-Ruiz, C. et al. Genomic-transcriptomic evolution in lung cancer and metastasis. Nature 616, 543-552 (2023), approved by an independent Research Ethics Committee, 13/LO/1546
Study protocol	https://clinicaltrials.gov/study/NCT01888601
Data collection	For the original clinical study, clinical and pathological data was collected from patients during study follow up - this period was a minimum of five years. Data collection was overseen by the sponsor of the study (Cancer Research UK & UCL Cancer Trials Centre) and takes place in hospitals across the United Kingdom. A centralised database called MACRO is used for this purpose. Recruitment started in 2014 across 6 sites (London, Leicester, Manchester, Aberdeen, Birmingham, and Cardiff) in the United Kingdom.
Outcomes	For the original clinical study, the main clinical outcomes were:<br>Disease-free survival (DFS) – measured from the time of study registration to date of first lung recurrence or death from any cause. Patients who do not have these events were censored at the date last known to be alive (including patients who developed a new

primary tumour that has been shown biologically to not be linked to the initial primary lung tumour).
Overall survival - measured from the time of study registration to date of death from any cause.

Plants

Seed stocks: N/A

Novel plant genotypes: N/A

Authentication: N/A

Flow Cytometry

Plots

Confirm that:

☒ The axis labels state the marker and fluorochrome used (e.g. CD4-FITC).

☒ The axis scales are clearly visible. Include numbers along axes only for bottom left plot of group (a 'group' is an analysis of identical markers).

☒ All plots are contour plots with outliers or pseudocolor plots.

☒ A numerical value for number of cells or percentage (with statistics) is provided.

Methodology

Sample preparation: Cells were seeded in 6-well plates at the densities stated above in 2 mL media 24 h prior to performing the assay. A final concentration of 1 µmol/L CellROX Green reagent was added to each well and incubated for 30 min at 37 °C, protected from light. Cells were then washed with PBS, harvested using 0.05% trypsin-EDTA and suspended in 1 mL of ice-cold Hanks balanced salt solution. The cell suspension was passed through a 35 µm filter and kept on ice prior to analysis.

Instrument: BD FACS Melody flow cytometer (488 nm laser and 527/32 bandpass filter; BD Biosciences).

Software: FlowJo

Cell population abundance: 20,000 single cell events were recorded per sample.

Gating strategy: Data was gated post-acquisition based on forward (FS) and side scatter (SS) profiles to include only single cell events and to exclude cellular debris.

☐ Tick this box to confirm that a figure exemplifying the gating strategy is provided in the Supplementary Information.
